# Novel SLCO2A1 mutations cause gender-differentiated pachydermoperiostosis

**DOI:** 10.1530/EC-18-0326

**Published:** 2018-08-30

**Authors:** Lijuan Yuan, Xihui Chen, Ziyu Liu, Dan Wu, Jianguo Lu, Guoqiang Bao, Sijia Zhang, Lifeng Wang, Yuanming Wu

**Affiliations:** 1 Department of Biochemistry and Molecular BiologyCenter for DNA Typing, Air Force Medical University, Xi’an, Shaanxi, People’s Republic of China; 2 Department of General SurgeryTangdu Hospital, Air Force Medical University, Xi’an, Shaanxi, People’s Republic of China; 3 Department of MicrobiologyAir Force Medical University, Xi’an, Shaanxi, People’s Republic of China; 4 Department of Biochemistry and Molecular BiologyAir Force Medical University, Xi’an, Shaanxi, People’s Republic of China

**Keywords:** primary hypertrophic osteoarthropathy, exome sequencing, solute carrier organic anion transporter family member 2A1, female atypical phenotype, hormone therapeutics

## Abstract

Primary hypertrophic osteoarthropathy (PHO) is a rare familial disorder with reduced penetrance for females. The genetic mutations associated with PHO have been identified in *HPGD* and *SLCO2A1*, which involved in prostaglandin E2 metabolism. Here, we report 5 PHO patients from four non-consanguineous families. Two heterozygous mutations in solute carrier organic anion transporter family member 2A1 (*SLCO2A1*) were identified in two brothers by whole-exome sequencing. Three heterozygous mutations and one homozygous mutation were identified in other three PHO families by Sanger sequencing. However, there was no mutation in *HPGD*. These findings confirmed that homozygous or compound heterozygous mutations of *SLCO2A1* were the pathogenic cause of PHO. A female individual shared the same mutations in *SLCO2A1* with her PHO brother but did not have any typical PHO symptoms. The influence of sex hormones on the pathogenesis of PHO and its implication were discussed.

## Introduction

Primary hypertrophic osteoarthropathy (PHO), which is also known as pachydermoperiostosis (PDP) (MIM 167100, 259100, 614441), is a rare familial disorder characterized by coarse facial features, gyrate scalp, clubbed nails and painful joint enlargement (1, 2, 3, 4). Castori *et al.* reviewed 204 published cases from 68 families with PDP and found that 37 families showed autosomal dominant inheritance while autosomal recessive inheritance was suggested in the remaining families (1). The pattern of segregation suggested sex-limited autosomal dominant inheritance, with reduced penetrance for females (5). The onset of the symptoms is usually around puberty with a male-to-female ratio of 9:1, and males are severely affected (2, 6, 7).

The first molecular genetic mutation related to PHO was identified in *HPGD* (MIM 601688) gene, which involved in the prostaglandin E2 metabolic pathway (8). *HPGD* encodes 15-hydroxyprostaglandin dehydrogenase (15-PGDH, EC 1.1.1.141), which mainly metabolizes prostaglandin E2 (PGE2). Solute carrier organic anion transporter family member 2A1 (*SLCO2A1*, MIM 601460) gene has also been shown to be associated with PHO (4). Mutations in *HPGD* or* SLCO2A1* result in loss of metabolizing capacity of PGE2 in PHO patients, which is supported by elevated urinary PGE2 level. Subsequently, a number of PHO or isolated, congenital clubbed nails cases were found to display *HPGD* or *SLCO2A1* mutations (9, 10, 11, 12, 13, 14, 15, 16, 17). Mutations in *HPGD* gene are responsible for autosomal recessive primary hypertrophic osteoarthropathy 1 (PHOAR1) (8) and mutations in *SLCO2A1* gene are responsible for autosomal recessive primary hypertrophic osteoarthropathy 2 (PHOAR2) (4).

**Figure 1:**
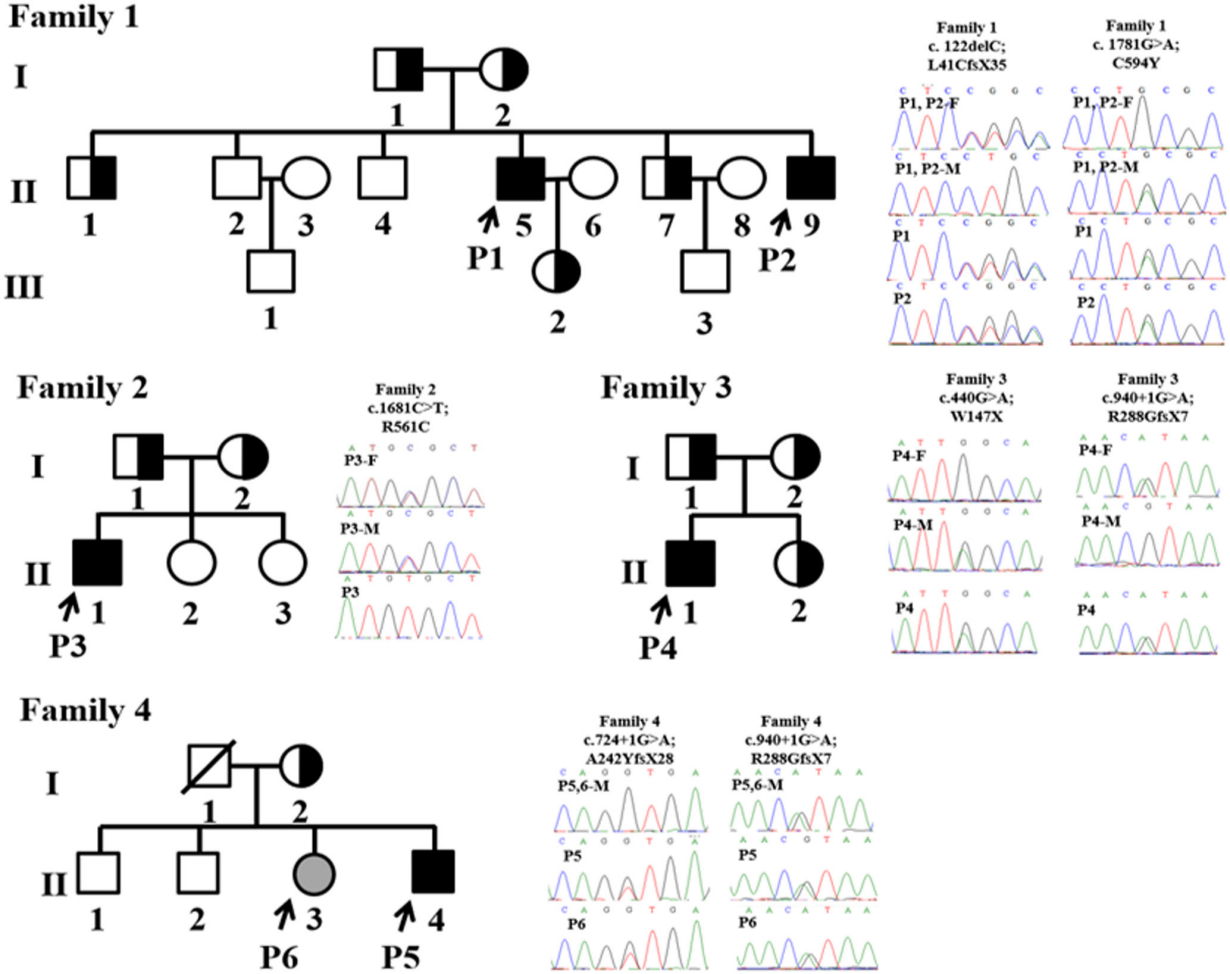
The pedigree chart of the four Chinese families affected by PHO and the location of the *SLCO2A1* mutations. Affected PHO patients are indicated by black symbols represent, unaffected individuals are indicated by open symbols and the asymptomatic mutation carriers are indicated by half-blackened symbols. Squares and circles indicate males and females, respectively. The arrows indicate the probands in each family. P1 and P2 had a heterozygous frameshift mutation in combination with a heterozygous missense mutation, and the father (P1, 2-F) and mother (P1, 2-M) were mono heterozygous carriers; P3 had a homozygous mutation and his father (P3-F) and mother (P3-M) were both mono heterozygous carriers; P4, P5 and P6 had compound heterozygous mutations, and their fathers and mothers (P4-F; P4-M; P5, 6-M; P5, 6-M) were mono heterozygous carriers.

Exome sequencing was performed to identify the causative genetic mutations in two male PHO patients (brothers) and their parents, siblings and other related family members (Fig. 1). Mutations were identified in one of the reported PHO causative gene *SLCO2A1*. More mutations of *SLCO2A1* were then identified in three other unrelated families. Moreover, a genotype-positive female was found without any PHO symptom, and the underlying mechanisms were discussed.

## Materials and methods

### Families and subjects

The four non-consanguineous PHO families for our research were recruited in the Dermatological Department of Xi Jing Hospital and Lanzhou Military Region Hospital. The 1, 2, 3, 4 families were all from northwest areas in China (Ningxia, Erdos, Lanzhou, and Xi’an, respectively). All patients were diagnosed by clinical manifestation and finger and long bone X ray. There was no family history of the PHO and no signs of the disease in other family members. Family 1 had a total of 14 individuals and two out of six sons suffered from PHO who were named as P1 and P2 (Family 1, II.5-P1; Family 1, II.9-P2). Family 2 had four asymptomatic members and the affected son was named as P3 (Family 2, II.1-P3). Family 3 composed of three asymptomatic members and the affected son was named as P4 (Family 3, II.1-P4). In Family 4, there were four asymptomatic members and the affected son was named as P5 (Family 4, II.4-P5). More detailed information was shown in Fig. 1. All subjects were of Chinese Han ethnicity. Three hundred eighty-four unrelated control individuals were recruited from Shannxi and Henan provinces. Venous blood samples were obtained from all showed individuals (5 affected and 23 unaffected). Genomic DNA was extracted from whole blood by using standard methods. Written informed consent was obtained from all participants. Our study was approved by the Ethical Review Boards of the Fourth Military Medical University and Lanzhou Military Region Hospital.

### Exome sequencing

Library preparation and exome sequencing: whole-exome sequencing was performed in two affected sons and their parents from Family 1. The qualified genomic DNA sample extracted from peripheral blood was randomly fragmented by Covaris and the size of the library fragments was mainly distributed between 250 and 300 bp. Then adapters were ligated to both ends of the resulting fragments. Extracted DNA was then amplified by ligation-mediated PCR (LM-PCR), purified and hybridized to the NimblegenSeqCap EZ Library for enrichment. Non-hybridized fragments were then washed out. Both non-captured and captured LM-PCR products were subjected to quantitative PCR to estimate the percentage of enrichment. Each captured library was then loaded onto Hiseq2000 platform. We performed high-throughput sequencing for each captured library to ensure that every sample met the desired average sequencing depth. Raw image files were processed by Illumina basecalling Software 1.7 for basecalling with default parameters and the sequences of each individual were generated as 90 bp pair-end reads.

Read mapping and variant analysis: The sequenced reads were aligned to the human genome reference (UCSC hg 18 version) using SOAP aligner (18). Duplicated reads were then filtered out. On the basis of SOAP aligner alignment results, Soaps software (19) was used to assemble the consensus sequence and call genotypes in target regions. The low-quality variations were filtered out by the following criteria: (i) quality score <20 (Q20); (ii) average copy number at the allele site >2; (iii) distance of two adjacent SNPs <5 bp and (iv) sequencing depth <4 or >200. For insertions or deletions (indels) in the targeted exome regions, reads were aligned to the reference genome using bwa (20). And indels were identified using GATK (21). Annotations of variants were made by in-house pipeline.

Analysis protocol for exome sequencing results: Since synonymous changes were far less likely to be causative, we filtered out synonymous mutations. Based on the hypothesis that the mutation underlying families with PHO should not be present in the general population, non-synonymous/splice acceptor and donor site/insertions or deletions (NS/SS/Indel) variants reported in the dbSNP129, HapMap Project (phase I, II, III), 1000 Genome Project (20100804 release) and in-house databases were removed. The functional impact of synonymous changes was predicted by SIFT software (version 4.0, http://sift.jcvi.org/). We further assumed that the two exome-sequenced patients should have the same NS/SS/Indel variants.

### Sanger sequencing

Sanger sequencing with customized primers was performed to determine the presence of the variants in all the clinically affected subjects and to screen the unaffected members in the family for co-segregation analysis. PCR reaction mixture contained 1 µL of each primer, 4 µL of dNTP, 5 µL of 10 × PCR buffer, 1.25 U TaKaRaTaq (TaKaRa Biotechnology (Dalian) Co., Ltd.) and 20–50 ng DNA template in a total volume of 50 µL. The nucleotide sequence of the amplified product was directly determined on AB 3730xl DNA analyzer (Applied Biosystems). Sequencing alignment was performed using BioEdit Sequence Alignment Editor (version 7.0.5.3).

## Results

### Typical PHO phenotypes presented in probands from non-consanguineous Han Chinese families

**Figure 2:**
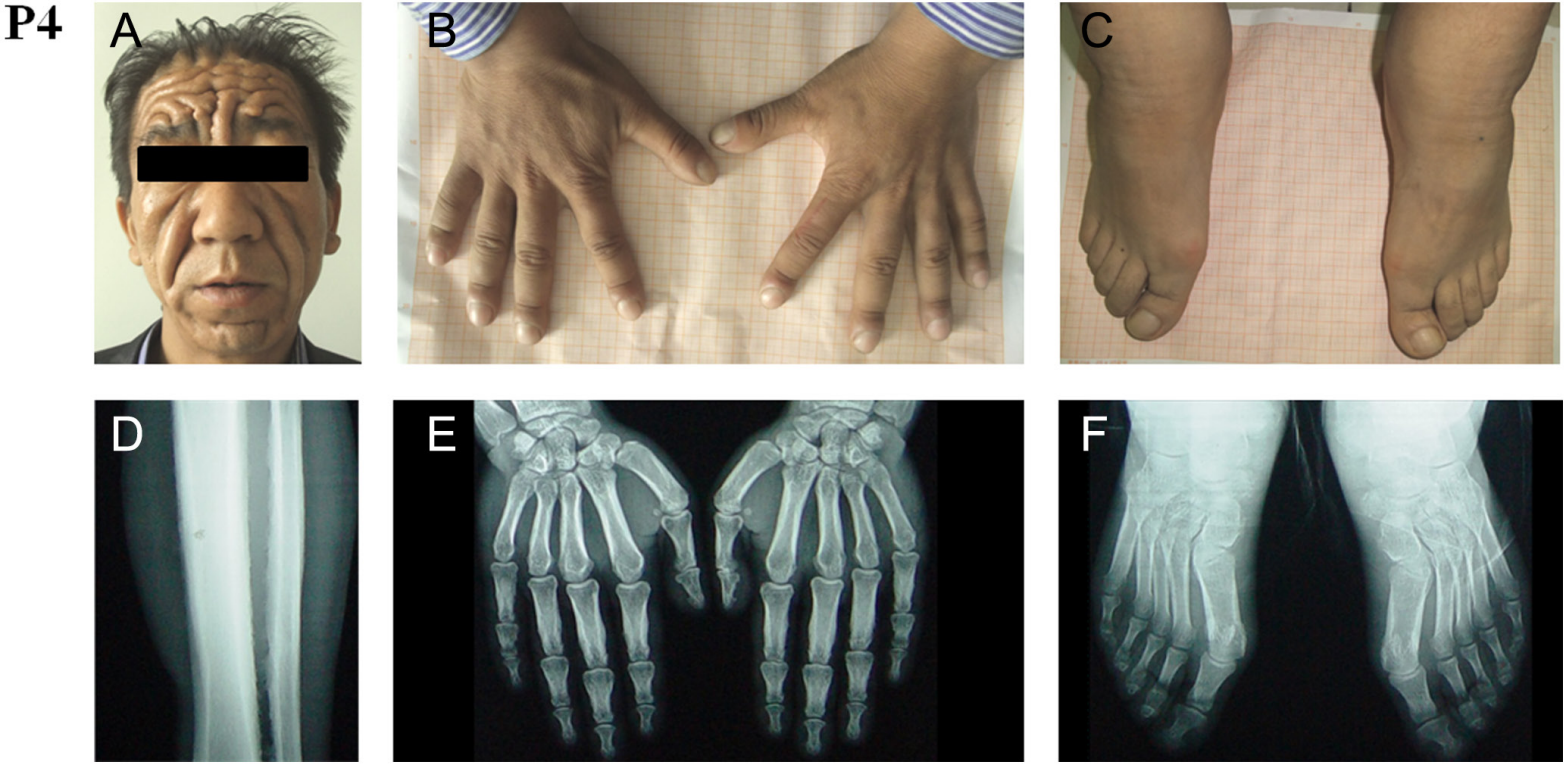
Clinical images of the affected individual: Family3, II.1-P4. The images showed the thickening and furrowing of facial skin (A) and the clubbing of fingernails and toenails (B and C). A radiograph of tibiofibula showed periosteal hyperostosis (D). A radiograph of the hands and feet showed cortical thickening and acroosteolysis (E and F). All images were published with permission from the affected individual.

Digital clubbing, swelling of the knees, periostosis and a progressive thickening and furrowing of facial skin were typically featured in Chinese PHO cases observed by us or others absent of any pulmonary pathology or developmental anomalies. Furthermore, the recessive inheritance was also typically observed in all reported Chinese PHO families. The pedigrees of the four unrelated Han Chinese families with PHO were shown in Fig. 1. All affected individuals had early childhood onset of typical PHO symptoms with clubbing, hyperhidrosis, periostosis, diaphyseal expansion and skin thickening. The disease was diagnosed in the affected persons with clinical and radiological criteria (Fig. 2, Table 1 and Supplementary Fig. 1, see section on supplementary data given at the end of this article). Patient 1 (P1, Family 1, II.5) and P3 (Family 2, II.1) mainly complained about their painful arthrophlogosis in combination with severe dermatological features. Although periostosis, thickened scalp and diaphyseal expansion were predominant in P4 (Family 3, II.1) and P5 (Family 4, II.4), neither had any symptom of joint. However, hyperhidrosis and seborrhea were more obvious in P4. Compared to his brother P1, P2 (Family 1, II.9) represented a rather lower grade of both dermatological and joint features. Physical examinations revealed that all PHO patients did not have other secondary hypertrophic osteoarthropathy, such as heart or lung abnormities. No other member from these four families had PHO symptoms.

**Table 1:** Clinical and molecular features of PHO patients.

Patient	P1	P2	P3	P4	P5
Pedigree labels	Family1, II.5	Family1, II.9	Family2, II.1	Family3, II.1	Family4, II.4
Gender	Male	Male	Male	Male	Male
Ages	43	37	25	33	38
Clubbing of fingers and toes	++	+	++	+	++
Thickened facial skin	+++	+	++	+++	+
Periostosis	++	++	++	++	+++
Thickened scalp	+	+	+++	++	+++
Hyperhidrosis	+++	+	+	+++	+
Seborrhoea	+++	+	+++	+++	+
Diaphyseal expansion	++	+	+	+++	+++
Arthralgia of large joints	+++	−	+++	−	−
Knee-joint effusions	+++	−	++	−	−
HPGD genotype	−	−	−	−	−
SLCO2A1 genotype	c.122delC (p.L41CfsX35); c.1781G>A (p.C594Y)	c.122delC (p.L41CfsX35); c.1781G>A (p.C594Y)	c.1681C>T (p.R561C); c.1681C>T (p.R561C)	c.440G>A (p.W147X); c.940+1G>A (p.R341HX2)	c.724+1G>A (p.A242YX28); c.940+1G>A (p.R341HX2)

### *SLCO2A1* mutations identified by exome sequencing and mutation screening in PHO

To identify the PHO causative genetic mutations in Han Chinese population, we performed exome sequencing in two PHO-affected sons and their parents from a non-consanguineous family (Family 1). In the exome capture result of four individuals from Family 1 (Supplementary Table 1), reads mapped to target region could reach from 41,664,251 to 44,651,698, covering at least 98.97% of target region, which was sufficient to pass our thresholds for calling SNPs and short insertions or deletions (Indels), with a rate of nucleotide mismatch below 0.3%. After identification of variants, we focused only on non-synonymous (NS) variants, splice acceptor and donor site mutations (SS) and short, frameshift coding insertions or deletions that were more likely to be pathogenic than other variants. The total number of SNP identified in P1 was 83,547. Among them, 8086 SNPs belonged to synonymous-coding mutation, 9389 SNPs in the UTR regions, 8955 missense SNPs and 92 nonsense SNPs (Supplementary Table 2). There were 7160 indels in coding regions or introns (Supplementary Table 3).

Since PHO is a rare disorder with a clear phenotype, the possibility of PHO patients sharing casual mutations with a healthy population is very low. Therefore, we compared all the detected variants in two probands with that of dbSNP132, HapMap project (phase I and II and III), 1000 Genome Project (20100804 release), YH database and two in-house databases from BGI and filtered out variants that were present in any one of these databases. For dbSNP132, HapMap project database, 1000 Genome Project database, we set a filtration threshold of 0.005 for minor allele frequency as we assume that these databases may include variants from causative allele carriers. After filtrations, only 266 rare variants were left (data not shown). Then, we further assessed the presence of co-segregation in all rare variants and identified only three potential PHO candidate genes with compound heterozygous variants, in which *HPGD* gene was not included. Considering deregulated systemic PGE2 levels are critical in PHO pathogenesis, the solute carrier organic anion transporter family member 2A1 (SLCO2A1), a prostaglandin transporter protein, was among the list. Sanger sequencing was performed to validate the identified mutations and further look for other mutations in this family and three other unrelated non-consanguineous Chinese families, which include 3 typical PHO patients and 11 asymptomatic carriers. The segregation analysis confirmed the *SLCO2A1* mutations co-segregated with the PHO in all these families. And again, no mutation of *HPGD* gene was detected among all subjects.

### Complete recessiveness for the *SLCO2A1* mutations

P1 and P2 (Family 1) harbored a heterozygous frameshift mutation (c.122delC; p.L41CfsX35) in exon 2 in combination with a heterozygous missense mutation (c.1781 G>A; p.C594Y) in exon 13 (Fig. 1). We also confirmed that three relatives (Family 1, II.1, II.7 and III.2) of these two probands were carriers of mono heterozygous mutations, while other relatives were unaffected. P3 (Family 2, -II.1) had a homozygous missense mutation (c.1681C>T; p.R561C) in exon 12 and the parents were all mono heterozygous mutation carriers (Fig. 1). P4 (Family 3, II-1) had a heterozygous missense mutation (c.440G>A; p.W147X) in exon 4 in combination with a heterozygous guanine-to-adenine transition at the invariant +1 position of the acceptor site of intron 7 (c.940+1G>A) of *SLCO2A1*, while I-1, I-2 and II-2 from this family were all mono heterozygous carriers (Fig. 1). P5 (Family 4, II.5) had a compound heterozygous of the guanine-to-adenine transition at the invariant +1 position of the acceptor site of both intron 5 (c.724+1G>A) and intron 7 (c.940+1G>A), while his mother (Family 4, I.2) was a mono heterozygous carrier and two brothers were all unaffected. To our surprise, the sister of P5 (P6, Family 4, II.3) was found to have the identical compound guanine-to-adenine transitions as P5 but did not show any typical symptom of PHO (Fig. 1).

All six genetic variants identified in the six PHO-affected individuals from four independent families were not somatic mutations but rather inherited. We did not detect any of the observed mutations in 384 unrelated and ethnically matched control individuals. These results further confirmed that the identified mutations in *SLCO2A1* were the independent cause of PHO.

### Conservation of the newly identified mutation site p.C594

**Figure 3:**
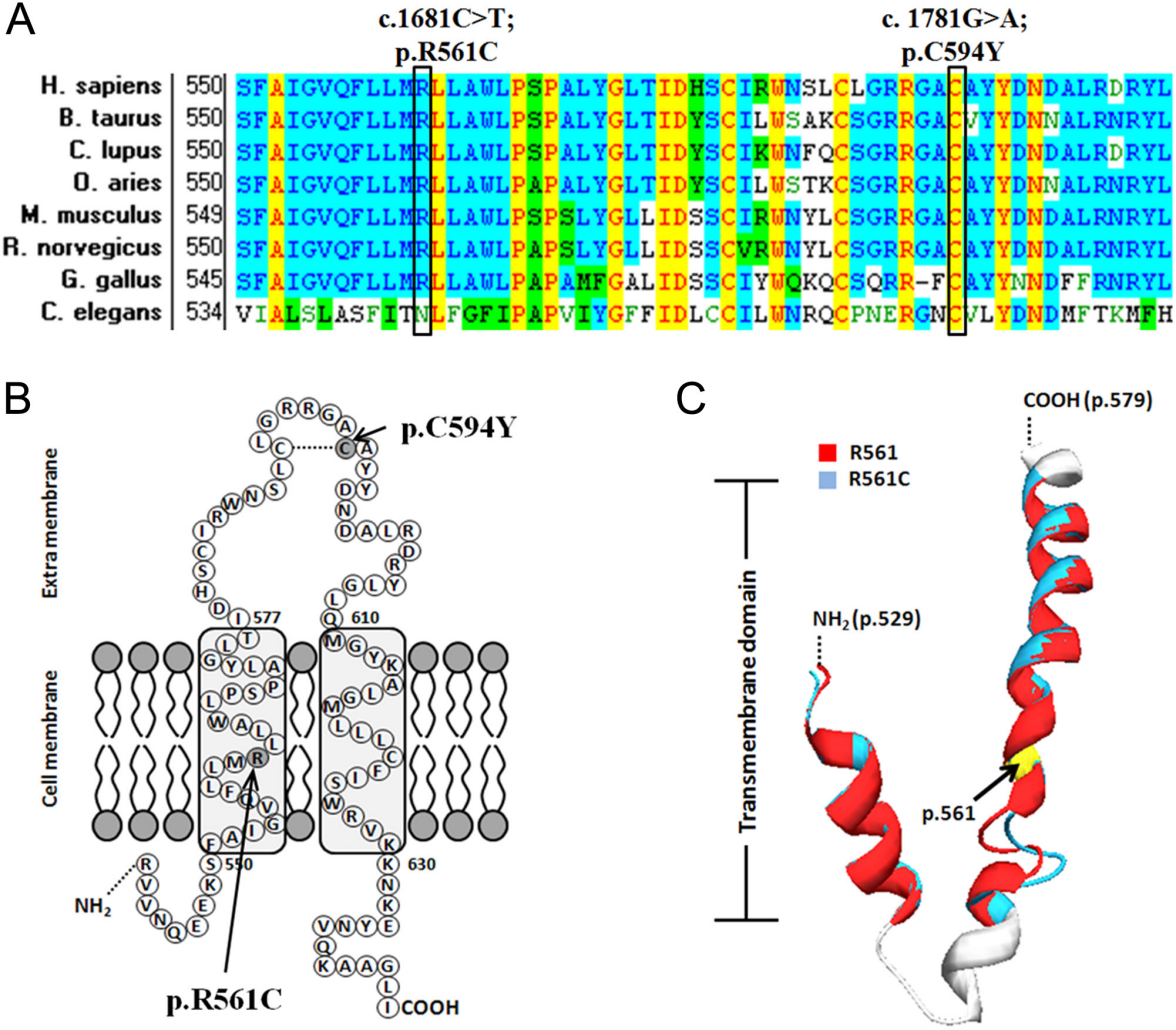
The orthologs and modeling of the p.R561C and p.C594Y missense mutations. (A) The alignment of the SLCO2A1 with the corresponding segments in eight species is shown. Both the p.R561C and p.C594Y missense mutations occur at the highly conserved position in the SLCO2A1. Amino acids marked with column are highly conserved among all shown species. Background color illustration: transparence: non-similar; light blue: conservative; yellow: identical. (B) Model of the prostaglandin transporter (*SLCO2A1*, the 11th and 12th transmenbrane regions) and location of missense mutations identified in this study. The homozygous mutation p.R561C is located within the 11th transmembrane region and the heterozygous p.C594Y mutation site is located in the extracellular loop between the 11th and 12th transmenbrane regions. p.C594 here was predicted to construct a Cys-Cys Zinc-finger motif loop with a nearby cysteine (p.C587). The mutations are indicated by arrows. The model was developed according to the NCBI protein database about SLCO2A1 (NCBI: NP_005621.2). (C) Modeling of the SLCO2A1 (529–579) p.R561C mutation. Superimposition of the WT SLCO2A1 R561 (red) and R561C mutation (blue). The R561 is localized in 11th transmembrane domain. The mutation twisted the loop structure between the adjunct 2 helix near the p.561 for about 90° rightwards.

Screening of *SLCO2A1* orthologs with the use of the NCBI HomoloGene database (http://www.ncbi.nlm.nih.gov/sites/entrez?cmd=Retrieve&db=homologene&dopt=MultipleAlignment&list_uids=38077) revealed that the p.C594 is highly conserved among human, chimpanzee, monkey, dog, cow, mouse, rat, chicken and frog (Fig. 3A). Cysteine 594 is at the extracellular loop between the 11^th^ transmenbrane and 12th transmenbrane domains in the C-terminal of SLCO2A1 and forms a disulfide bond with cysteine 587 (22, 23) (Fig. 3B and Table 2).

**Table 2:** Mutations identified in PHO causative SLCO2A1.

DNA change	Exon/intron	Putative amino acid change	Location (NP_005621.2)	Premature stop	Pathogenicity	Families (*for consanguineous)							SIFT score	SIFT prediction
						*	*	*						
122delC	2	L41CfsX35	Transmenbrane (region 1st)	N.D.^a^	Compound heterozygous				**+**					
440G>A	4	W147X	Extracellular (between 3,4)	N.D.	Compound heterozygous						**+**			
724+1G>A	5–6	A242YX28	Extracellular (between 5,6)	N.D.	Compound heterozygous							**+**		
940+1G>A	7–8	R341HX2	Transmenbrane (region 7th)	N.D.	Compound heterozygous						**+**	**+**		
1681C>T	12	R561C	Transmenbrane (region 11th)	No	Homozygous					**+**			0.00	Damaging
1781G>A	13	C594Y	Extracellular (between 11,12)	No	Compound heterozygous				**+**				0.00	Damaging

^a^Not defined.

### Damaging effects of the mutations predicted by the SIFT algorithm

SIFT was an analysis tool primarily applied to predict the impact of an amino acid substitution caused by NS SNP on the function of a given protein (24). Here, we analyzed our newly found amino acid substitution (AA) p.C594Y and the reported mutation p.R561C and both got a score of 0.00 (Table 2). The SIFT prediction scores ranged from 0 to 1 and a substitution would affect the protein function when the score fell below 0.05.

### Malfunction of p.R561C indicated by structure modeling

A molecular model of SLCO2A1 was constructed with the SWISS-MODEL server and Swiss-Pdb Viewer (25, 26) with the template structure 3o7q retrieved from PDB. Although the sequence identity was only 11.77%, the amino acids from 529 to 579 were covered (Supplementary Table 4), which including part of the 10th and the full 11th transmembrane domains of SLCO2A1. The mutation R561C was localized in the 11th transmembrane domain. The loop structure between the adjacent helix was twisted rightward for about 90° by the p.R561C mutation, which was predicted to impair the transport of the prostaglandin (Fig. 3C). Moreover, the cationic amino acid was essential for binding and transport of PGs (23, 27). The amino acid residual cysteine 594 could not be covered by any of the PDB data. However, this highly conserved cysteine was located in the extracellular loop that participated in the formation of the receptive pocket of prostaglandin binding (Fig. 3B).

### A female with identical genotype with a PHO patient was exempt from clinical symptoms

**Figure 4:**
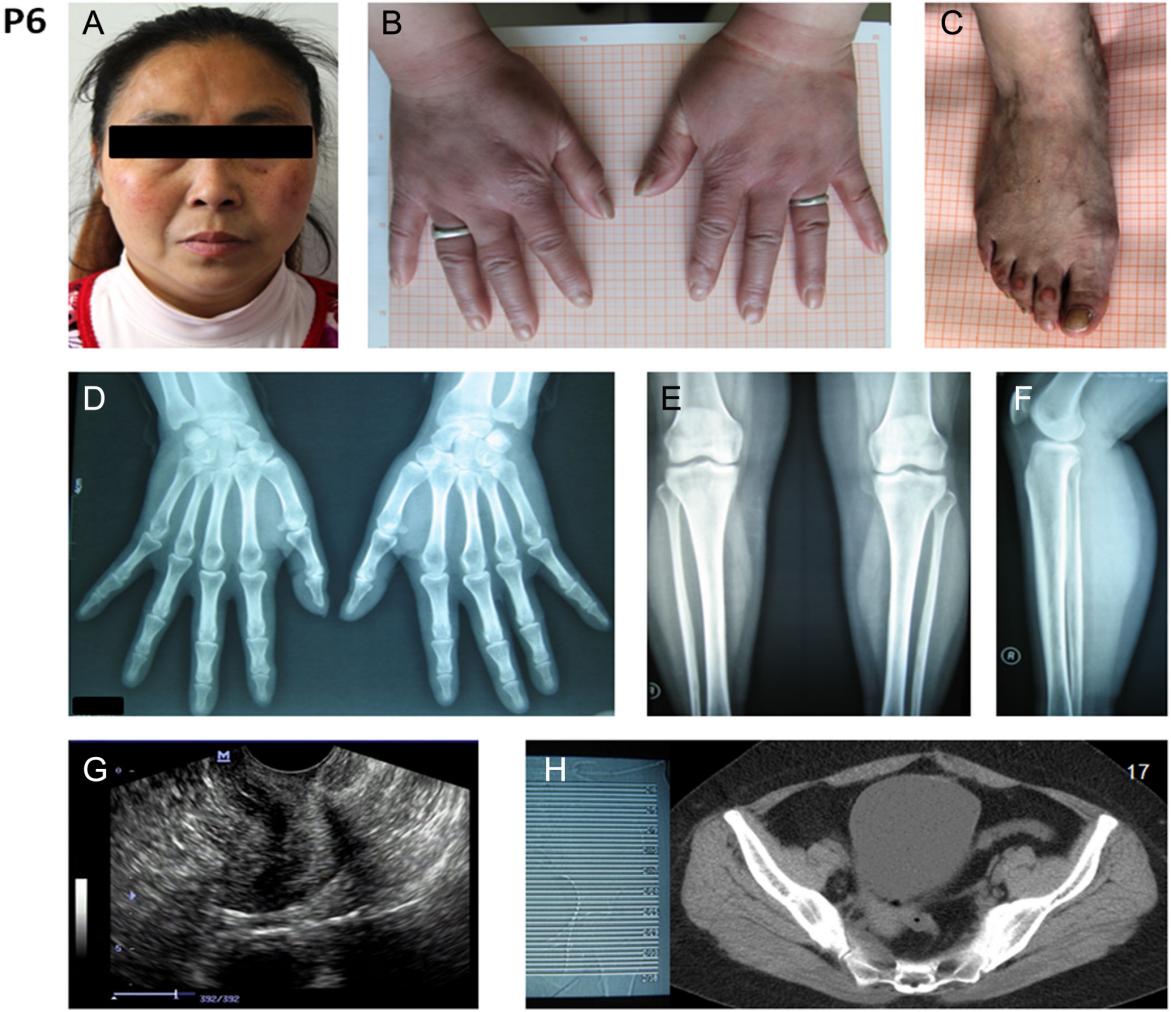
Clinical images of the unaffected individual: Family4, II.3-P6. The images showed no thickening and furrowing of facial skin; and the clubbing of fingernails or toenails (A, B and C). A radiograph of tibiofibula, hands and feet show no sign of periosteal hyperostosis (D, E and F). Gynecological B-ultrasound examination showed invisibility of both ovaries (G). CT screen is also hard to detect both ovaries (H). All images are published with permission from the individual.

Although bearing the identical mutations with her brother P5, a 40-year-old female (P6, Family 4, II.3 in Fig. 1) did not present the typical PHO symptoms (Fig. 4A, B, C, D, E and F). Physiological and radiological examinations revealed no clubbing of fingers and toes, no thickened scalp and no periostosis. She claimed for a premature menopause at age 37 years. To verify her complaints, gynecological B-ultrasound examination and CT scan were performed. The premature ovarian atrophy was diagnosed by invisibility of both ovaries (Fig. 4G and H) accompanied by the atrophy of uterus. Serum sexual hormones were then detected by radioimmunoassay. Estradiol and progesterone levels were low (Table 3), which demonstrated the character of menopause. We further examined her hormone levels of pituitary–thyroid axis, pituitary–adrenal axis and pituitary–gonadal axis. The normal thyroid-stimulating hormone (Table 4) and adrenotrophin (Table 3) levels and the elevated level of the follicle-stimulating hormone (Table 3) illustrated the normality of her pituitary endocrine functions, which indicated that the degeneration of sex organs was the primary feature of females carrying compound heterozygous mutations of *SLCO2A1*.

**Table 3:** Hormone analysis.

Items	Result	Unit	Reference value
Testosterone	<10.0	ng/dL	Adult female: 0–80; adult male: 225–1158
Estradiol	22.5	pg/mL	Menopause female: 10–25; adult male: 25–52
Progesterone	0.3	ng/mL	Menopause female: 0.1–1.52; adult male: 0.10–0.75
Follicle-stimulating hormone	31.3	mlU/mL	Menopause female: 13.0–89.5; adult male: 1.5–6.5
Luteinizing hormone	23.7	mlU/mL	Menopause female: 13.0–115.0; adult male: 1.3–8.3
Prolactin	13.2	ng/mL	Adult: 0–25
Cortisol	15.8	µg/dL	Adult: 4.2–22.3
Growth hormone	0.9	ng/mL	Adult female: 0.5–4.8; adult male: 0.5–2.3
Thyrotrophin	1.7	µIU/mL	Adult: 0.3–6.0
Adrenocorticotrophic hormone	6.8	pg/mL	5.1–32.0

**Table 4:** Thyroid hormone analysis of P6.

Items	Result	Unit	Reference value
FT3	3.6	pg/mL	2.45–7.04
FT4	18.1	pmol/L	9.05–25.5
T3	1.3	ng/mL	0.78–2.2
T4	88.8	ng/mL	45–125
TSH	2.1	µIU/mL	0.3–5.0
TPOAb	2	%	<20%
TgAb	5	%	<30%
TG thyroid globulin	7.2	ng/mL	1.7–55.6

The female patient (P6) also complained a history of dysmenorrhea and menorrhagia in adolescence but did not have a history of gastrointestinal problems. Shortly after marriage at 18 years, she conceived and beared a healthy son. Thereafter, an irregular menstrual was persistent until the menopause. A hypoferric anemia was complained and the bone marrow histology showed medullary erythrocytic hyperplasy (data not shown).

## Discussion

The present work presented exome sequencing data of two probands from the same family. After SNP filtration, co-variants filtration and the co-segregation analyses, three potential PHO causative genes with compound heterozygous mutations were identified and one of them (*SLCO2A1*) was verified by Sanger sequencing within this family and in three other unrelated non-consanguineous Chinese families.

*SLCO2A1* encodes a prostaglandin transporter that is a member of the 12-membrane-spanning superfamily of transporters. This gene is located on 3q21 of the human chromosome and the full-length transcript contains 14 exons. The encoded protein SLCO2A1 (in abbreviation) is also alternatively named prostaglandin transporter (PGT), which mediates the active transepithelial uptake of prostaglandins against a concentration gradient (28). It has been shown to transport several prostaglandins, including PGD2, PGE1, PGE2, PGF2a and PGH2 (22, 28, 30). Carrier-mediated uptake followed by cytoplasmic oxidation is important for the removal of local prostaglandin (PG) signaling, and dysfunction either of which may otherwise be pathogenesis for certain sign of disease such as PHO (3, 4, 8, 12, 16, 31).

Various kinds of mutation types were detected among the five individuals with PHO in this study, which included a heterozygous **frameshift** mutation (c.122delC), a heterozygous **nonsense** mutation (440G>A), two heterozygous **splice-site mutations** (c.724+1G>A; c.940+1G>A), a heterozygous **missense** (p.C594Y) and a homozygous **missense** (p.R561C) in *SLCO2A1*. Both the p.R561C and p.C594Y missense mutations are at highly conserved positions and likely to be functionally damaging. Besides our functional structure prediction, R561 is among the three highly conserved charged amino acids, namely E78, R561 and K614, critical for PGE2 transport activity (23, 27). Site-directed mutagenesis of R561 in the 11th transmembrane domain almost completely aborted the binding and transporting activity of PGs of rat PGT (22, 32). The p. C594Y mutation might alter the structure of the extracellular Cys-Cys Zinc-finger motif loop (22, 23) and disrupt the receiving pocket of prostaglandin. This mutation occurred in combination with a heterozygous single-base deletion (c.122delC), which resulted in a frameshift and a premature termination codon (p.L41CfsX35) in the 1st transmembrane domain of SLCO2A1. The combination of heterozygous c.122delC (p.L41CfsX35) and c.1781 G>A (p. C594Y) mutations resulted in PHO while either of them caused PHO symptoms individually. Similarly, P4 from Family3 carried a heterozygous c.440G>A mutation, which would result in a premature termination codon (p.W147X) in the extracellular loop between the 3rd and 4th transmembrane domains. The mutation occurred in combination with a heterozygous c.940+1G>A splice-site mutation, which would result in the skipping of exon 7, leading to a frameshift and a premature termination codon (p. R288GfsX7). P5 and P6 from Family 4 also had a heterozygous c.940+1G>A splice-site mutation in combination with another heterozygous splice-site mutation c.724+1G>A, which resulted in the skipping of exon 6, leading to a frameshift and a premature termination codon (p. A242YfsX28).

Many of the single gene inheritance diseases could trace to a solo or few defined mutations; however, PHO-associated *SLCO2A1* variants consisted of a relatively broad spectrum of mutations. More than 50 different *SLCO2A1* mutations have been identified in patients with PHO (33, 34, 35, 36, 37, 38, 39, 40, 41, 42, 43, 44, 45), which are located in different exons or at splicing sites, led to changes to different functional domains or truncation of the protein. Within the six *SLCO2A1* mutations identified in the current study, four of them caused premature stop of translation, which included one nonsense mutation, one frameshift mutation and two splice-site mutations. The other two were missense mutations, which led to change of highly conserved amino acids and impairment of PG transporter function. The promiscuous mode of mutations in this disease's causative gene may indicate an inheritance of acquired spontaneous gene mutations raised from definite genetic, physiological and environmental factors.

All PHOAR2 patients reported here carried compound heterozygous or homozygous mutations in *SLCO2A1* while mono heterozygous mutations did not cause PHO symptoms. It was consistent with the recent reports that the loss-of-function *SLCO2A1* mutations were responsible for most PHO cases in China (4, 31, 33, 36, 37). Moreover, the recessiveness was also reported in a variety of Europe, Latin America, North Africa and East-Asia populations (34, 38, 39, 40, 41, 42, 43). PHO patients with *SLCO2A1* mutations did not show failure of postnatal ductus arteriosus closure but developed a late-onset phenotype that was most prominent after puberty, suggesting a different pathogenic mechanism from *HPGD* mutations.

Interestingly, a female who shared identical mutations with her PHO brother did not have PHO symptom but presented anemia, very early menopause and ovary atrophy with altered hormone levels. The recent studies also showed that fewer females were affected by pathogenic* SLCO2A1* mutations (9, 31, 44). Two females aged 34 and 19 years with homozygous pathogenic *SLCO2A1* mutations were both without typical PHO symptoms but the elder individual was affected with mild finger clubbing and severe transfusion-dependent anemia (39). Although the symptom was suggested to be related to myelofibrosis, which was a newly defined feature of PHO, there was no direct clinical data for the typical macrocytic anemia of myelofibrosis. Our clinical data of the female carrier (P6) did not have myelofibrosis features. Niizeki *et al*. reported that women patient may develop late-onset PDP symptoms with atypical phenotype (45). Considering the importance of prostaglandin transporter in the regulation of prostaglandin action in the menstrual cycle of human female (22, 46, 47), we postulated that some hormones might protect female from the severe symptom of PHO in skin and bones, which hinted a potential hormone therapeutics for the temporary unaffected preadolescent male probands. Meanwhile, sex hormones would be responsible for the difference in the prostaglandin metabolism in women of reproductive age and in women in menopause. The altered sex hormone levels might contribute to the late-onset symptoms in females (45). The deregulation of menstrual cycle might be a new clinical feature for women *SLCO2A1* mutations carrier.

**Figure 5:**
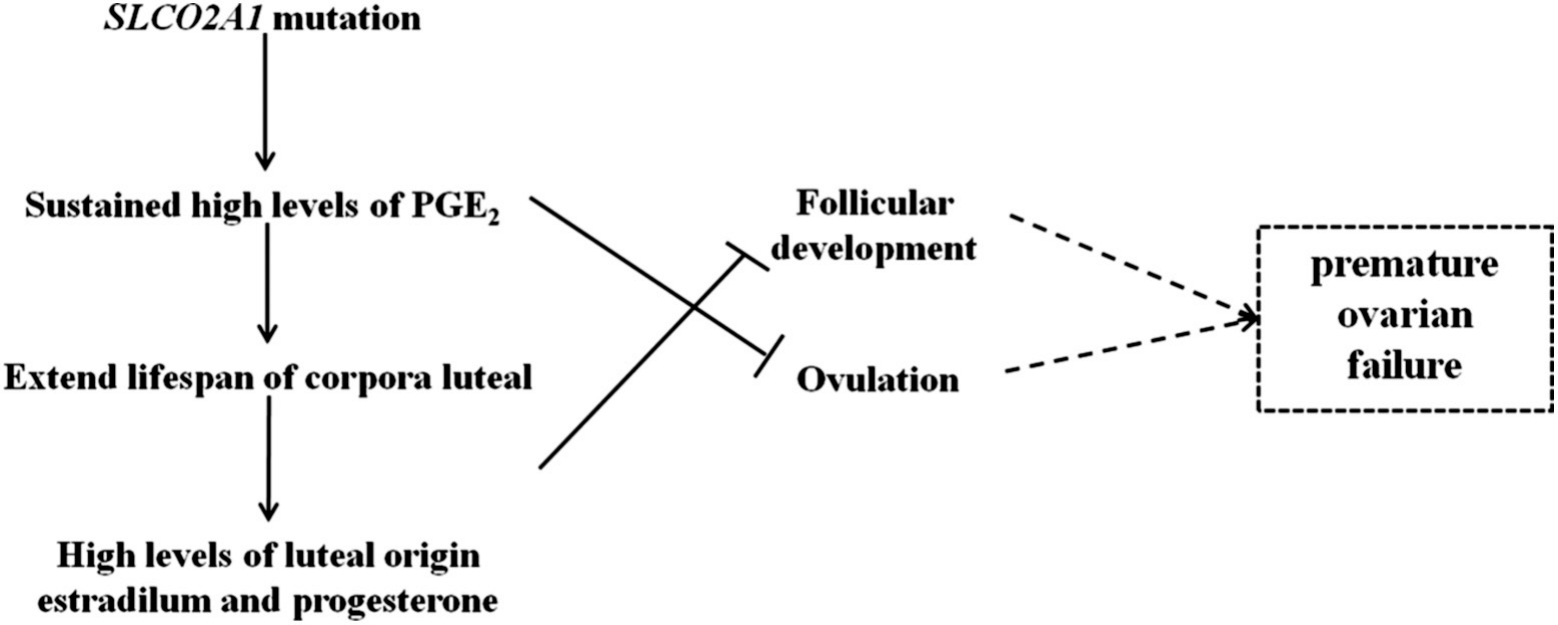
Predicted model for SLCO2A1 mutation-induced premature ovarian failure. SLCO2A1 mutations lead to a high local concentration of PGE2 by the dysfunction of PG transporter. PGE2 is luteotropic and could continually maintain the high levels of luteal origin estrogen and progesterone, meanwhile, extend the lifespan of corpora luteal. However, estrogen, of luteal origin, is responsible for the suppression of follicular development and PGE2 could inhibit the frequency of ovulation. The SLCO2A1 mutations caused long-lasting inhibition of ovarian physiology cycle may induce the premature ovarian failure.

As for pathogenic *SLCO2A1* mutation carriers, the metabolism of PGE2 and PGF2α would be inhibited as the plasma PGE-M raised nearly 2-fold and the urinary PGE-M or PGE2 could raise nearly 10-fold (4). The blockade of PG transporter in females may lead to higher local concentration of both PGE2 and PGF2α, which would inevitably disturb the PG regulation of the menstrual cycle (48). The uncontrolled levels of luteotropic PGE2 would maintain high levels of luteal origin estrogen and progesterone (49) while inhibit the oxytocin-induced release of PGF2α from the endometrium (22). Taken together, the* SLCO2A1* mutations induced long-lasting high levels of PGE2, leading to abnormal levels of estrogen and progesterone that are harmful to the ovarian physiology (Fig. 5). The PHO-causing *SLCO2A1* mutation-carrying female indeed presented the premature ovarian failure and the extremely early menopause with altered hormone levels.

As for why *SLCO2A1* mutant female could be free of the typical PHO symptoms (digital clubbing, pachyderma, periostosis and *et al*.) presented in males, it might involve the differences of sex hormones of males and females as well as the deregulation of PGE2 or even PGF2α. The onset of PHO symptoms usually appears around puberty (9) – the period of the development of sexual characteristics and changes of hormone levels (50). One of the main differences between males and females is the regulation of different sex hormones, such as the distinctive elevated level of testosterone in male and periodical rise and fall of estradiol and progesterone (menstrual cycle) in female. This difference of hormone levels was responsible for the gender difference of asthma (51, 52), rhinitis (52) and depression (53) among other conditions. Estrogen has been shown to increase the risk of pulmonary hypertension (54) but promoted fat metabolism and protected against obesity (55). High androgen-to-estrogen ratio was shown to be associated with higher risk of esophageal adenocarcinoma (56) but androgen could block estrogen-induced proliferation of breast cancer cells (57). Similarly, the raised level of female hormones at puberty might somehow inhibit the detrimental effects of PHO-causing *SLCO2A1* mutations and protected female carriers from PHO symptoms. Accordingly, two scenarios could be raised for potential hormone therapy for the unaffected preadolescent male *SLCO2A1* mutation carriers. First, the females are always in low concentration of testosterone from the adolescent compared with males. The sharply raised concentration of testosterone in adolescent male may synergize with the dysregulation of PG to cause PHO pathogenesis. Thus, temporary administration of androgen antagonist may prevent the onset of PHO. Second, the females always have high concentrations of either estradiol and progesterone or FSH and LH, which may protect females from the onset of PHO. Therefore, these female hormones may be adopted for preventing PHO in *SLCO2A1* mutation-carrying adolescent males.

## Supplementary Material

Supporting Figure 1

Supporting Table 1

Supporting Table 2

Supporting Table 3

Supporting Table 4

## Declaration of interest

The authors declare that there is no conflict of interest that could be perceived as prejudicing the impartiality of the research reported.

## Funding

The present study was supported by the National Natural Science Foundation of China (grant no. 81671476 and 81502424).

## Author contribution statement

Lijuan Yuan, Xihui Chen and Ziyu Liu finished the data analysis and wrote the paper; Dan Wu and Lifeng Wang prepared the figures and tables; Jianguo Lu, Guoqiang Bao and Sijia Zhang reviewed and edited the manuscript; Yuanming Wu conceived and designed the study. All authors read and approved the manuscript.
