# Supplementary figures and images for: Novel SLCO2A1 mutations cause gender-differentiated pachydermoperiostosis
Source: Endocr Connect. 2018 Aug 30;7(11):1116–28. doi: 10.1530/EC-18-0326 (PMC6223238; doi:10.1530/EC-18-0326)

**P1**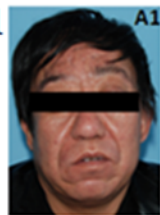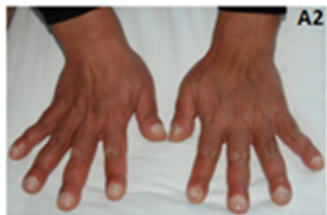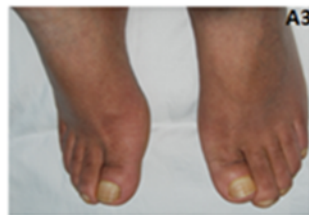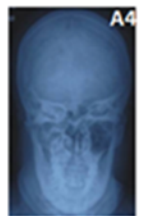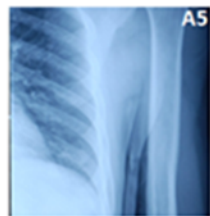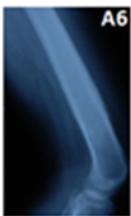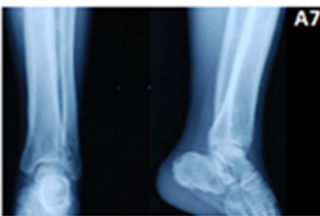**P3**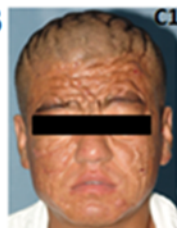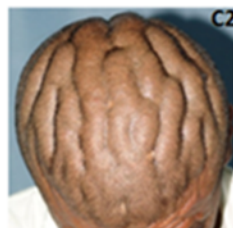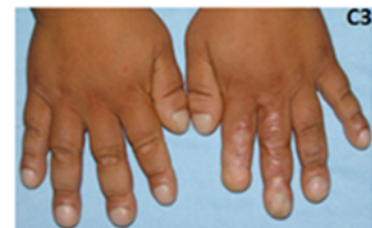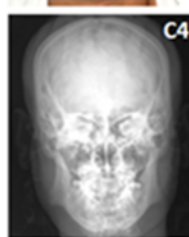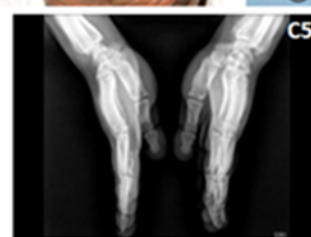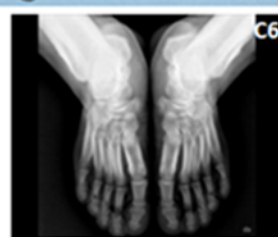**P2**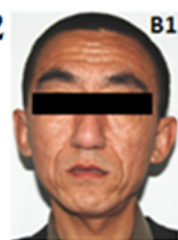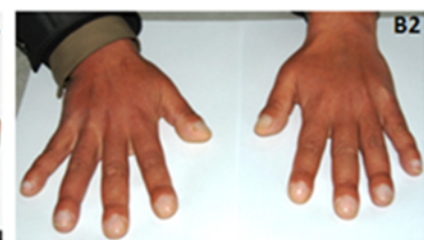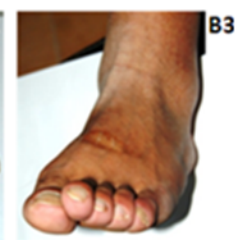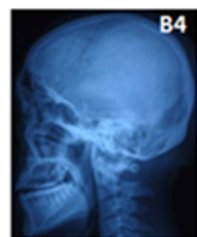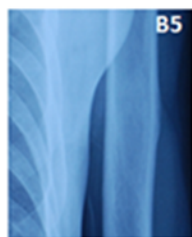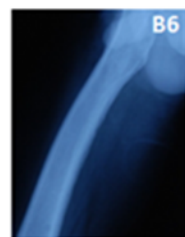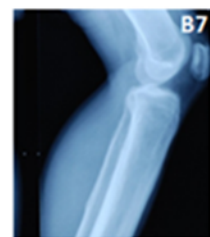**P5**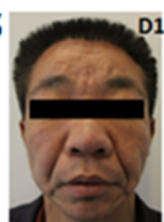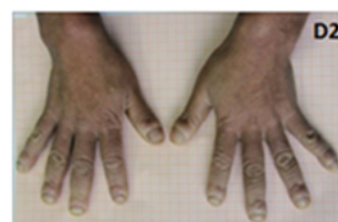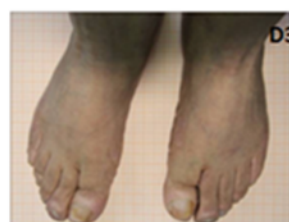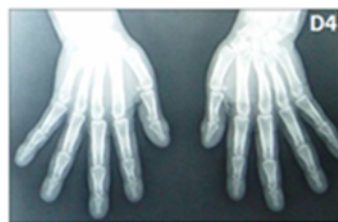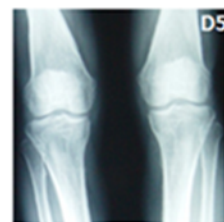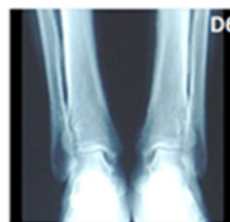

Supplement: Supporting Figure 1 [file ec-7-1116-s001.pdf]
