# Supplementary material for: Novel SLCO2A1 mutations cause gender-differentiated pachydermoperiostosis
Source: Endocr Connect. 2018 Aug 30;7(11):1116–28. doi: 10.1530/EC-18-0326 (PMC6223238; doi:10.1530/EC-18-0326)
Supplement: Supporting Table 1 [file ec-7-1116-t001.pdf]

Supplemental Data

Table S1. Overview of data production

**Exome Capture Statistics**	**P1, 2-F**	**P1, 2-M**	**P1**	**P2**
Target region (bp)[a]	44234141	44131461	44234141	44234141
Raw reads	68385084	66027242	72306004	70327526
Raw data yield (Mb)	6155	5942	6508	6329
Reads mapped to genome	65047149	62578970	68417785	66714870
Reads mapped to target region[b]	44087635	41664251	44651698	43645787
Data mapped to target region (Mb)	3105.9	2936.9	3155.5	3090.1
Mean depth of target region(X)	70.2	66.5	71.3	69.9
Coverage of target region (%)	99.0	99.0	99.1	99.0
Average read length (bp)	90	90	90	90
Rate of nucleotide mismatch (%)	0.3	0.3	0.3	0.3
Fraction of target covered >=4X (%)	97.4	97.2	97.5	97.4
Fraction of target covered >=10X (%)	94.2	93.7	94.4	94.0
Fraction of target covered >=20X (%)	87.2	86.3	87.7	86.8
Capture specificity (%)[c]	69.5	68.1	66.8	66.9
Reads mapped to flanking region[d]	10402508	9451190	9822056	9380012
Mean depth of flanking region(X)	25.0	23.2	24.4	23.5
Coverage of flanking region (%)	97.4	97.1	97.6	97.4
Fraction of flanking covered >=4X (%)	88.1	86.3	87.3	86.3
Fraction of flanking covered >=10X (%)	66.8	63.7	64.9	63.3
Fraction of flanking covered >=20X (%)	42.7	39.8	41.2	39.6
Fraction of unique mapped bases on or near target (%)	84.6	82.4	80.3	80.2
Duplication rate (%)[e]	2.8	3.1	3.3	3.0
Mean depth of chrX(X)	47.8	85.8	48.6	47.6
Mean depth of chrY(X)	82. 7	-	91.5	90.3
GC rate (%)	44.9	44.8	45.2	45.1
Gender test result	M	F	M	M

a: Target regions here refer to the regions that are actually covered by the designed probes.

b: Reads mapped to target regions are reads that within or overlap with target region.

c: Capture specificity is defined as the percentage of uniquely mapped reads aligning to target region.

d: Flanking region refers to regions +/-200 bp on both sides of each target region.

e: PCR duplicates would have the same start and end for both mates, which rarely occurs by chance.

Duplication rate is the fraction of duplicated reads in raw data.
