# Supplementary material for: Novel SLCO2A1 mutations cause gender-differentiated pachydermoperiostosis
Source: Endocr Connect. 2018 Aug 30;7(11):1116–28. doi: 10.1530/EC-18-0326 (PMC6223238; doi:10.1530/EC-18-0326)
Supplement: Supporting Table 2 [file ec-7-1116-t002.pdf]

Table S2. Summary of SNPs for exome capture sample

Categories	P1, 2-F	P1, 2-M	P1	P2
Number of genomic positions for calling SNPs[a]	127061576	126755209	127061576	127061576
Number of high-confidence genotypes[b]	118847805	117999674	118747916	118356058
Number of high-confidence genotypes in TR	43381914	43316860	43421103	43387817
Total number of SNPs	84841	83064	83547	82499
Missense	9000	9229	8955	8996
Readthrough	62	63	65	61
Nonsense	110	96	92	103
Splice site[c]	2073	2115	2098	2083
Synonymous-coding	8100	8345	8086	8166
5-UTR	6872	6732	6798	6655
3-UTR	2621	2540	2591	2525
Intron	54340	52299	53267	52304
Intergenic	1663	1645	1595	1606
Hom	38856	37038	38928	37879
Het	45985	46026	44619	44620

a: Genomic positions for calling SNPs are defined as all the positions in *.cns file, which include capture target regions and its 200bp flanking regions.

b: Consensus genotype with quality score of at least 20.

c: Intronic SNPs within 10bp of exon/intron boundary.
