# Supplementary material for: Novel SLCO2A1 mutations cause gender-differentiated pachydermoperiostosis
Source: Endocr Connect. 2018 Aug 30;7(11):1116–28. doi: 10.1530/EC-18-0326 (PMC6223238; doi:10.1530/EC-18-0326)
Supplement: Supporting Table 3 [file ec-7-1116-t003.pdf]

Table S3. Summary of InDels for exome capture samples

sample	P1, 2-F	P1, 2-M	P1	P2
Total number of indels	7168	7149	7160	7109
Frameshift	242	251	250	235
Cds-Indel	200	206	206	184
Splice site	395	389	415	408
5-UTR	626	612	620	599
3-UTR	254	257	244	279
Intron	5314	5312	5297	5281
Promoter[a]	32	25	26	24
Intergenic	105	97	102	99
Hom	3004	2902	3012	2989
Het	4164	4247	4148	4120
Total insertion	3389	3347	3371	3358
Total deletion	3779	3802	3789	3751
Ins-coding[b]	205	207	205	205
Del-coding[c]	237	250	251	214

a: Promoters are located near the genes they regulate, on the same strand and typically upstream 1000bp towards the 5' region of the sense strand.

b: Insertion in coding sequence.

c: Deletion in coding sequence. These two(2, 3) cases can cause potential frame-shift mutations.
