# Supplementary material for: Novel SLCO2A1 mutations cause gender-differentiated pachydermoperiostosis
Source: Endocr Connect. 2018 Aug 30;7(11):1116–28. doi: 10.1530/EC-18-0326 (PMC6223238; doi:10.1530/EC-18-0326)
Supplement: Supporting Table 4 [file ec-7-1116-t004.pdf]

Table S4. Model information of SLCO2A1

Model information（SWISS-MODEL）	
Modelled residue range:	529 to 579
Based on template:	[3o7qA] (3.14 Å)
Sequence Identity [%]:	11.77
Evalue:	1.50e-7
QMEAN Z-Score:	-3.1
